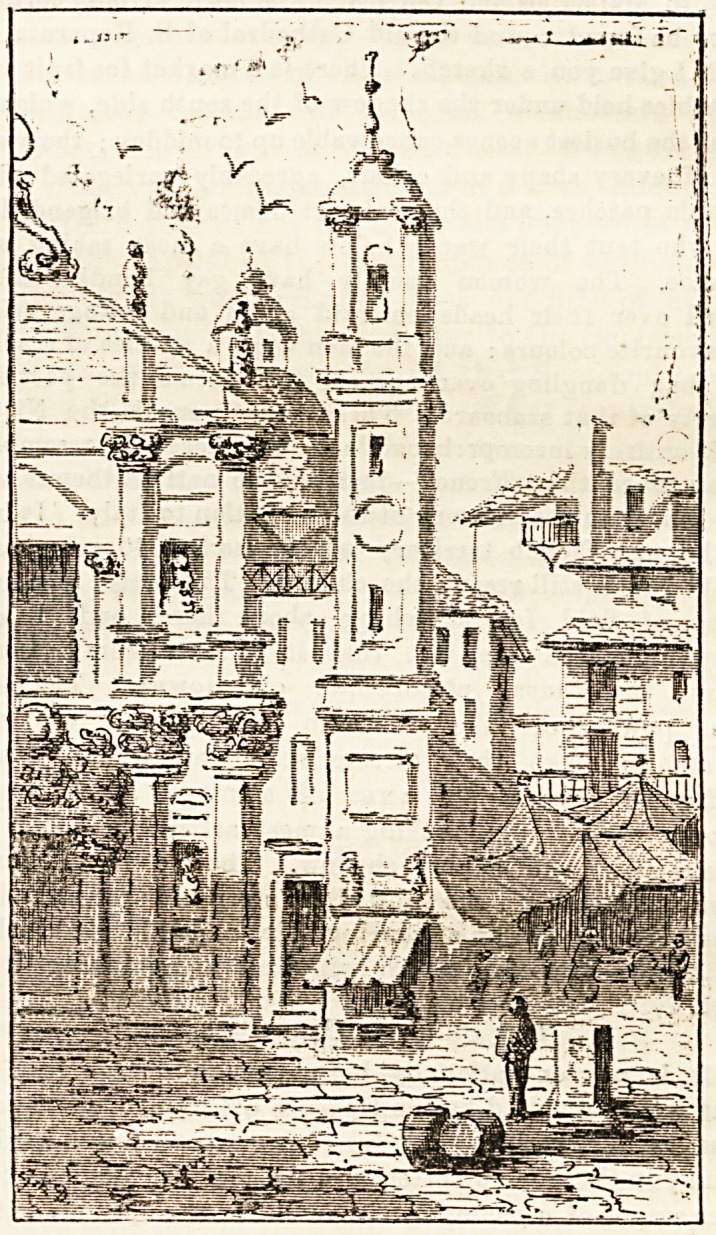# "The Hospital" Nursing Mirror

**Published:** 1898-12-31

**Authors:** 


### "The Hospital" Nursing Mirror.

Being the Nursing Section of "The Hospital."

[Contributions for this Section of "The Hospital" should be addressed to the Editor, The Hospital, 28 & 29, Southampton Street, Strand, London, W.C., and should have the word "Nursing" plainly written in left-hand top corner of the envelope.]

### News from the Nursing World.

### OUR CLOTHING DISTRIBUTION.

Our clothing distribution has been very successful this year. In the first place, our kind helpers have been liberal, both as regards the number and quality of their gifts. The garments have been beautifully made of good material, whilst to our list of other donors must be added the name of our old and valued contributor, Madame Monchablon, who with her friends sent a very large number of things indeed. All one afternoon was taken up in sorting and packing them for their destination, and once more we chose such hospitals as were most in need of help. Eleven bulky parcels were the result, and their destination were as follows: Charing Cross Hospital; Westminster Hospital; King's College Hospital; the Alexandra Hospital for Hip Disease; the North-Eastern Hospital for Children, Hackney Road; the Great Northern Hospital; the West Ham Hospital; the East End Mothers' Home; and the West London Hospital. Some were distributed in person, but others had to be despatched to those far outlying by carrier. We hope to let our workers know from the matrons themselves how gratefully these gifts were received. Those we visited were delighted, and the sisters charmed. The two children's hospitals had two parcels apiece, one containing dolls and balls. We regret that Miss Rosetta Mason's kind gift has been received too late for this distribution. We shall keep it as a nest egg to our next year's work.

### A CRITICISM AND A SUGGESTION.

The imperative need of trained nursing in the treatment of tropical diseases is constantly being brought home to us by the object lessons so amply furnished by current history. Criticising the proposal made in the columns of the *Journal of Commerce*, to establish educational training homes in London and Liverpool for instructing in the nursing of tropical disease, Mr. J. J. Lamprey suggests that, as the demand for nurses is immediate, it would be possible to establish a volunteer system, by which women already experienced in general nursing could, after an additional course in fever nursing, be enrolled under some such title as "The West African Nurses' Association" or "The Volunteer Nurses' Association for Service Abroad." The following is an outline of the terms suggested during employment: Ages, 25 to 35; engagement for one year at first, with good salary and outward and return passage, and a six months' holiday at the usual rate of salary in England. Further terms of service as desirable, and finally a pension at the end of ten years' service. It is certainly most desirable that nurses going to such climates should be well paid during their arduous task, and provided for when disabled, as we fear too many will be by the dangers inseparable from the work.

### MORE NURSES FOR THE WEST LONDON HOSPITAL.

On December 7th the Princess Louise visited the West London Hospital to open the new wing, and spent a long time there. She expressed the greatest interest in everything she saw. This new wing consists of two handsome wards, one over the other, named the "Mary Adelaide" (a women's surgical), and the "Devonshire" (a men's medical) respectively, the former after the late Duchess of Teck, and the latter after the President, the Duke of Devonshire. The first patients will be admitted on January 2nd, and everything is being put in readiness for them. These new wards necessitated raising the number of the nursing staff to 42. Sister Tuffnell, who was trained and who spent six years altogether at the London, is in charge of the "Devonshire" ward; and Sister Wells, who was trained and attached to the South Devon and East Cornwall for a period of eight years, is in charge of the "Mary Adelaide" ward. Pending the building of a new nurses' home, a couple of old houses belonging to the hospital have been put in order and afford comfortable and roomy accommodation. The matron regrets that she cannot yet give each nurse her own room; nevertheless, the apartments allotted to two nurses (and no more) are large and airy. The sisters' rooms in the new wing leave nothing to be desired, and a convenient closet adjoining enables all toilet paraphernalia to be hidden away to their owners' great satisfaction.

### ASTON NURSES' HOME.

The organisation of the Aston Nurses' Home, the outcome of the Diamond Jubilee celebration, has progressed rapidly. Although the home will not be formally opened until the annual meeting in February next, a superintendent and two district nurses are already at work, the nomination cards have been distributed, and arrangements have been made for the nurses to work in connection with the relief association. The last provision appears admirable; it frees the nurse from the undesirable position of almoner, which is fatal to her influence for good, and at the same time places her in touch with an association which will supplement her work with necessities. Lady Warwick has promised to perform the opening ceremony.

### AN INTERESTING CAREER.

It is with much regret we announce the death of Miss Amy Castilla, M.D., said to be the first lady resident medical officer of a general hospital in the southern hemisphere. Of late years Miss Castilla has been in private practice in Melbourne. She was one of the first women graduates of the University of Melbourne. Her ancestry is also worthy of notice. Her grandfather, a Spanish refugee, fled to London and became secretary to the younger Pitt, and her father was among the very earliest settlers in Melbourne. Miss Castilla began her professional career at the Alfred Hospital in that city, where she was a nurse before she qualified for the medical profession, and became resident medical officer at St. Vincent's Hospital.

### HOUSE-TO-HOUSE COLLECTIONS.

THE ladies of Liverpool render substantial help to the Hospital Saturday Fund by organising a systematic house-to-house collection on its behalf. The Lady Mayoress and other ladies of good social position take an interest in the work, and the honorary secretaries deserve much credit for the energy and tact with which they conduct their operations. The report of last year's work is most encouraging, for the returns were beyond the collectors' most sanguine anticipations. In 1895 the amount collected was £316; this year it was £737. A noteworthy and encouraging feature has been the eagerness displayed by the working men to contribute, which certainly suggests the thought that the possible reason for the public lukewarmness in the matter of small subscriptions is the absence of any organisation founded on businesslike lines for collecting them. The evils connected with street collections are self-evident, and no one can regret the universal disfavour with which they are now regarded; but nothing can be said against a regular systematic house-to-house collection by accredited and acceptable members of a properly constituted association for the purpose; it seems a worthy and dignified method of securing the necessary money to support a universally approved and needed public charity.

### NEW NURSES' HOME AT WANDSWORTH.

A LARGE nurses' home, capable of accommodating 75 nurses, is being built in connexion with the Wandsworth and Clapham Union Infirmary. It occupies a site in the northern part of the Infirmary grounds, and is to be connected with the main building by means of an open covered way. There will be four floors, with corridors running the full length of the structure on each. The main staircase, bath-rooms, lift, &c., will be placed in the centre, and a couple of external fire-escape staircases will be built in convenient positions. The night nurses' quarters will be on the top floor, and there will be a large recreation-hall on the ground floor, next to the entrance-hall. Separate apartments will be provided for an assistant matron, as well as a sitting-room for the sisters. The building has progressed so far that the foundation-stone was laid in the middle of December by Mrs. J. Breward Neal, wife of the medical superintendent.

### WHAT THE RED CROSS SOCIETY SPENT IN THE SOUDAN.

THE help extended to the wounded in the Soudan campaign by the National Aid Society covered a period of three months (August to October), and the cost amounted altogether to £2,726; £300 of this sum was expended on the Egyptian troops and £200 in the relief of the wounded dervishes. The efficient manner with which the work of conveying the sick down the river was carried out by Colonel Young won the encomiums of Lord Cromer, and the nurses have every reason to congratulate themselves upon the recognition their services obtained.

### NEW NURSES' HOME AT LEEDS.

AN important addition to Leeds Infirmary is the new Nurses' Home, which was opened early this month by Mr. James Stables. The home is constructed to afford accommodation for 52 nurses, and provides each with a separate bedroom. On the ground floor is a sisters' sitting-room and another for nurses, as well as a handsome recreation-room. There are also a small visitors' reception-room, a writing-room, and a kitchen for the preparation of light refreshments, more substantial viands being served in the dining-room in the principal building. A bicycle-shed for 30 machines has been made in the courtyard. The home and infirmary are connected by a subway. The heating is effected by hot-water radiating coils and the lighting by electricity. The erection of this residence, which will add so materially to the comfort of the nurses of the infirmary, has been brought about by the handsome benefaction of £6,000 from Mr. Stables, who performed the opening ceremony.

### ANOTHER NURSING FUND.

IT is almost impossible to gauge accurately the enormous increase of district nursing associations. Scarcely a week passes without the news of some new association, big or little, being brought to our notice. The manner of establishing two nurses at Bilston and one at Bradley, both in the neighbourhood of Birmingham, must be very satisfactory both to the committee and nurses. A number of gentlemen residing in Bilston have subscribed £600 for the inauguration and maintenance of the three nurses, under conditions that secure this boon to the district for five years.

### SHORT ITEMS.

A NURSE, whom the late German Emperor delighted to honour for her valuable services during the Franco-Turkish War, was found dead at her writing-table on the 21st inst. at her home at Melling, Lancaster. This was Madame C. Rumpff, who for the last eleven years has lived alone, occupying herself with writing serial stories from the material provided by her campaign experiences. Madame Rumpff was 64 years of age.—Nurse Hall, who has been in the employ of the Newbury Board of Guardians for several years, has just had her salary increased by £10. She now has £40 a year. —The Runcorn Board of Guardians have invited the Yarmouth Board to join them in urging the Local Government Board to establish institutions in which nurses may be especially trained for service in such infirmaries. The Yarmouth Guardians have replied that they will support the memorial.—After Christmas a number of wards at the Victoria Park Hospital, E., will be reopened, and the nursing staff will therefore be increased. Our readers will probably remember that the matron has made arrangements by which a satisfactory one-year certificate can be exchanged for a three-year certificate on the completion of the remaining two years of service.—Each invalid soldier setting out for home from the Soudan on board the troopship "Jelunga" was provided by the Army Aid Society with two suits of pyjamas, a can of marmalade, a can of jam, and a bottle of scent.—The Local Government Board consider that the estimate of the proposed nurses' home and hospital at Sheffield—computed at £11,560—is too high for the accommodation provided. The Guardians have sent a copy of the letter containing these observations to the architect and are now awaiting his reply.

### Nursing in Enteric Fever.

By WARREN G. WESTCOTT, L.R.C.P.Lond., M.R.C.S.Eng., Resident Medical Officer, Chichester Infirmary.

IN enteric fever, perhaps to a greater extent than in any other acute specific disease, a successful issue depends upon skilful nursing. From first to last the patient is threatened by many serious complications, numerous and varied as in no other morbid state, which although unavoidable even in the most carefully managed case, can still be considerably lessened as regards frequency of occurrence, and if occurring, the accompanying dangers much mitigated by the care of a nurse who knows her patient's condition, and the precautions to be observed to guide it to a happy issue. Equal attention should be bestowed in every case, even if apparently mild in nature, as the ultimate course of events is uncertain; many which begin with slight symptoms later suffer severely from complications and frequently relapse, probably because thought to be not severe, and so subjected to insufficient care in matters of absolute rest and regulated diet. Whilst the nurse's first thought should always be for her patient's welfare, she must at the same time be ever mindful of all measures calculated to prevent dissemination of infection; the strictest personal and general hygiene should, therefore, be observed. To consider first what these matters are. She is nursing a case of infectious disease, and consequently needs to remember that it may be transmitted either directly from the patient or indirectly by means of articles which have been contaminated, infection of others, including herself, being far more likely to take place by the latter than by the former method; indeed the chance of such by mere proximity to the patient is extremely small. To diminish all risk she should keep her hands scrupulously clean and nails short. After handling anything that has been in use, as the bed-pan, enema apparatus, and so forth, after changing linen and bedclothing, and on the completion of all such work as sponging the body or cleansing the mouth, she should well wash her hands in hot water, using plenty of soap and a nail brush, and then rinse them in an antiseptic solution, as biniodide of mercury (1 in 1,000), perchloride of mercury (1 in 500), or carbolic acid (1 in 40); by these details the hands will be effectually rid of all infected matter. As to precautions for the safety of others, all linen, &c., which has been soiled should be promptly removed and immersed in a solution of carbolic acid (1 in 20) for at least half an hour, afterwards boiled for two hours or more; the bed-pan should, when not in use, be filled with some disinfectant, one of the above, or, better still, an acidulated solution of perchloride of mercury (1 in 500) being employed. On being taken to the patient it should still contain sufficient to cover the stool, and on removal from the bed should be surrounded by a cloth wrung out in some of the same and tranferred to the lavatory for the medical officer's inspection if ordered or deemed necessary; otherwise, immediately disposed of. The pan should finally be well scalded with plenty of hot water and again filled with the solution and kept ready for future requirements. The bed-pan sink, or, in the case of private houses, the pan of the water-closet which is used for the purpose, must at the same time be thoroughly flushed with a good volume of water and treated with the disinfectant. If this plan be efficiently carried out, chance of general infection falls to a minimum.

Now as regards the general management. The temperature should be taken four-hourly and registered on a suitable chart, together with the frequency of the pulse and respiration, as also each action of the bowels and the amount of urine passed. The pulse in the early stage of the affection is somewhat peculiar, of very low tension, and easily compressible. The dicrotic pulse: This to an inexperienced finger is apt to cause some misapprehension, as the radial artery at the wrist seems to pulsate just double the number of times that it really does. In order to avoid error make lighter pressure than usual with the fingers when seeking to ascertain its rate. In counting the number of respirations per minute watch the movements of the chest rather than place the hand upon it; the former can usually easily be done, the latter, especially in children, tends to disturb the respiratory rhythm; in cases, however, where the breathing is very shallow, the hand must be in contact with the chest wall. In charting an action of the bowels indicate by some sign, as, for instance, the letter H beneath it if there has been hæmorrhage. At first sight it may appear to be unimportant to note the quantity of urine passed, but retention is by no means an uncommon symptom in those cases in which extreme prostration is prominent, so that unless the amount be known such a state is likely to be overlooked. A specimen should be reserved for the medical officer's inspection if any abnormality seems to be present.

The body should be carefully sponged with tepid water to which a small amount of spirit has been added, say ℥i to the pint, every morning and evening, taking care to cause as little disturbance as possible. By this, in addition to cleansing the skin, the patient is much refreshed, and after the evening sponging sleep is frequently induced. A careful watch must always be kept for threatening bed-sores, as indicated by redness over parts subjected to pressure, *e.g.*, the shoulders, buttocks, and heels; if there be any sign of such the skin should be hardened by some form of spirit, and the patient may with advantage be placed upon a water-bed, and change of position encouraged as often as is thought desirable. Bed-sores are prone to occur in elderly and debilitated subjects, and in those cases in which the patient passes everything in the bed the parts ought to be kept as clean as possible, and, as indeed in all cases after an action of the bowels, the perineal region should be washed with perchloride of mercury solution (1 in 1,000); as a general rule, the dorsal decubitus is to be preferred, and always on any aggravation of of the abdominal symptoms, but if hypostatic congestion of the lungs supervene, the lateral position, right and left alternately, is desirable. The mouth should be cleansed at least three times during the day and oftener if necessary. Great gentleness must be employed in those cases in which the tongue and adjacent parts are much fissured. Absorbent cotton-wool made into swabs and soaked in a solution of potassium permanganate (gr. v. ard ℥i) is the best means of effecting the purpose. The teeth should be treated in a similar manner, or a soft indiarubber tooth brush may be used. In children, instead of the above, one may use the glycerine and borax of the Pharmacopœia. With the passing away of headache delirium often appears, and when in this condition the patient frequently attempts to leave the bed unless watched by the nurse. As such a proceeding, if it happens, entails much risk, every precaution must be taken to prevent it.

The amount of bronchial catarrh present varies greatly. In adults it seldom calls for treatment; in children, on the other hand, it is often more urgent. The application of a "pneumonia jacket" is usually all that is required. Bronchitis may occur as a symptom, and is then treated by the usual means.

Abdominal pain and tenderness are not, as a rule, much complained of during the early part of the affection. Children, however, not uncommonly suffer such quite at the beginning, and, if so, warmth may be applied to the abdomen as by cotton wool. Fomentations give much relief.

(*To be continued.*)

### Nursing in Paris Hospitals.

### C.—THE NURSING SISTERS.

### VIII.—THEIR PRESENT DOMAIN: PRIVATE HOSPITALS.

SINCE the eviction of the Sisters from most of the public establishments their partisans have been very active in establishing private hospitals to put under their charge. These hospitals are a practical answer to the challenge of the laicisers in the great debate in the Chamber of Deputies on December 20th, 1890, when laicisation had its final triumph, and the official seal put upon it by M. Constans. I have before referred to the special discourse given by him on that occasion as Minister of the Interior. The Minister declared himself personally extremely well disposed toward the Sisters, having seen them in their nursing work not only in Paris, but in many fields, even " five thousand leagues from Paris." Nevertheless he proclaimed a *non possumus* in the matter of ministerial intervention in the Paris laicisation. The law of 1849 on the Assistance Publique, extended in 1851, gave the absolute right to Paris to manage its own hospitals as it pleased. M. Constans twitted M. Paul de Cassagnac with not applying the same principle of local rights claimed in the matter of religious education for country communes to this question in regard to Paris itself. Dr. Desprès disputed the assertion that Paris favoured laicisation, but M. Constans pertinently inquired how the opinion of Paris could be better known than by the election of the Municipal Council. Moreover, being asked if he had not power to reprove the director of the Assistance Publique, he replied that though he certainly had such power, he had no such right to do so unless the director had misconducted his office, and it was no misconduct to select such nurses as the director chose so long as the chosen ones executed the work properly.

The sting of this famous discussion on December 20th, 1890, was, however, in regard to the qualifications of the Sisters, M. Constans himself, while posing as their friend, giving them a nasty stab by recalling that in 1871 at Berck-sur-mer (as was well known, though he did not name the hospital) seven children were killed by the mistake of a Sister in administering medicine. Again in the debate, M. Calvinhac (who, in view of subsequent development, made a rather absurd financial plea that laicisation had been economical) had a passage of arms with M. Baudry d'Asson as to whether the Sisters even knew how to bandage wounds. M. Calvinhac first stated that they never do any bandaging. M. d'Asson asserted that he himself had been bandaged by a Sister, whereupon M. Calvinhac backed down a little, but said the Sisters mostly are ignorant of the function. The outcome of the debate was that the partisans of the Sisters were put on their mettle. They felt that they had lost the battle for the time being as regards the official hospitals, but that every effort must be made to show that the Sisters are quite able to conduct any modern hospital with the latest improved methods, and, moreover, that as many Parisians as possible should be induced to use private hospitals conducted by the Sisters, and to boycott the public hospitals while the Sisters themselves were under a boycott therein. Several of the private hospitals in charge of the religious nurses had already been started, as if to prepare for the coming storm, but after 1890 there was a new impetus given to the movement.

These new private hospitals are under various orders of Sisters, some of whom were in the public hospitals before the laicisation, and others, although nursing nuns elsewhere, were not so employed in Paris.

In the first class is the Hahnemann Homœopathic Hospital at Neuilly, with 25 beds (which really dates from 1870), in charge of the Sisters of Charity. The Daughters of St. Vincent de Paul have also charge of the model Hôpital Saint Joseph, in the Rue Pierre Larousse at Plaisance, near the barrier at the back of the Montparnasse Cemetery. The Saint Joseph Hospital was founded in 1884, and has practically served as a model for the new Boucicaut Hospital described in the last article. The Hôpital Saint Joseph has 205 beds, for both men and women, with underground communication between the wards the same as I have described at Boucicaut. Another institution in charge of the Sisters of Charity is the Petit Hôpital Saint Michel, founded in 1888, with 30 beds (10 for women and 20 for men), situated in the Saint Lambert quarter, in the little Avenue Sainte Eugénie off the Rue Dombasle, near the Porte de Versailles, and also near the great orphan asylum of Saint Vincent. The Saint Vincent Sisters also serve the important Isaac Pareire dispensary at Levallois-Perret.

The Augustinians of the Hôtel Dieu have charge of the Hôpital Nôtre Dame de Bons Secours at Montrouge, in the Rue des Plantes, 72 beds, 36 for each set. This hospital was established in 1887, by Abbé Carton, the vicar of the great church of Saint Peter of Montrouge, in the extreme southern centre of modern Paris.

The new hospitals under the care of Sisters who were not evicted by laicisation include the Jules Gonin dispensary and surgical hospital at the famous northern Barrière de Clichy, with 24 beds, in charge of the no less famous modern Paris order of the Sisters of Saint Joseph, from their home across the river in the Rue St. Jacques. The Sisters of Saint Joseph are of worldwide fame as teaching nuns, but here dabble a little in nursing work. They were founded in 1816 by Mother Javoukey to ameliorate the social wounds of the Revolution and the Napoleonic wars, and have since spread into many lands. The Sisters of Saint Joseph of Cluny have also charge of the International hospital in the Rue de la Santé, founded in 1892 by the great surgeon of Saint Louis Hospital, the late Dr. Jules Péan, with 45 beds.

The modest order of the Franciscans of Calais, founded in 1852 by the Bishop of Arras by uniting seven small establishments in his territory, have charge of the appropriately-named Hospital of Saint Francis, with 40 beds, in the Boulevard Saint Marcel, near the Salpetrière.

The Sisters of the Presentation of the Holy Virgin, established in 1684 in Beauce at Sainville, near Chartres, but uprooted during the Revolution, and re-established in Touraine at Saint Symphorien-les-Tours in the later days of Napoleon by Marie Poussepin, who have a noble hospital and war nursing record in the past, have charge of a second homœopathic hospital (Hôpital Homeopathique Saint Jacques), founded in 1871 in the Vaugirard district, and situated in the Rue de Voluntaires, with 60 beds. The Sisters of the Presentation also conduct the shop girls' retreat at Vanves, in the south-western suburb, founded in 1884.

The great Vendean order of the "Daughters of Wisdom," founded in 1703 by the missionary De Montford, have charge of the Maison Sainte Emilie, with 21 beds, instituted in 1890 at the beautiful suburban Clamart. The little modern order of the Sisters of Our Lady of the Cross, founded at Murinais, near Grenoble, in 1832, have charge of the Sainte Anne Maison de Santé at Chatillon, partly devoted to penitent Magdalens.

Lastly, Baroness Mackau and Madame de Vatimesnil and their mother, the Comtesse Maison, founded in 1885 at the opposite end and very unbeautiful suburb of Levallois Perret the Hôpital Nôtre Dame de Perpetual Secours with 52 beds (26 for each sex), in charge of the third order of Dominican nuns.

Besides the regular hospitals the houses of nursing sisters who go into private residences gratuitously, sometimes refusing even their food, are too numerous in Paris to enumerate.

We may take it that the private hospitals are likely to run the public institutions a great rivalry in the favour of the well-to-do classes of society, and perhaps of all classes. EDMUND R. SPEARMAN.

### Christmas Entertainments.

### Christmas Carols at St. Thomas's Hospital.

On Tuesday afternoon the wards at St. Thomas's Hospital were once more resplendent in their Christmas dress, and outside, from the Embankment, the long lines of windows, glowing with many colours, gave a bright and festive air to the great building. Tea, as usual, was served for visitors in the dining-room at the Nightingale Home at five o'clock, and presently the long procession of "Nightingale probationers," in their mauve and white striped uniform, started off, two and two, on their yearly progress round the many wards. Three or four carols are usually sung in each ward, so that the entertainment takes a considerable time; but it is one that is evidently thoroughly enjoyed, alike by the singers and their audience. The ward decorations at St. Thomas's are always a pleasure to see, and the Sisters must be heartily congratulated on the successful result of their efforts in that direction this year. In the surgical wards the edict has gone forth that drapery is for the future forbidden on these occasions, but gaily-coloured Chinese lanterns and myriads of fairy lamps, combined with plenty of glossy green palms and ferns in Doulton pots, make the most effective decoration possible, and the wards, one and all, were really brilliant on Tuesday evening. Lamp shades, chiefly cunningly contrived of paper in various shades and tints, were a great feature, and of wonderful ingenuity and effect. At the entrance of one ward the initials of the surgeons were arranged in fairy lamps on a ground of dark green art muslin, and in another the name of the ward glowed red through the glass above the door, cleverly illuminated by tiny lamps. The fresh voices of the "Nightingales" sounded well in many a favourite old carol, not forgetting "Good King Wenceslaus" and not a few very pretty new ones. The singers, according to old custom, finished with "Auld Lang Syne," which was taken up and concluded with fervour by students and visitors, and applause and congratulation followed the nurses down the long corridor. Concerts in the wards and entertainments of various kinds go on this week, and on Saturday the great event is to be the Christmas tree in Victoria, the children's ward, and a "doll competition," of the details of which readers of The Hospital will be interested to hear next week. Children's special hospitals are usually flooded with toys and dolls and other youthful delights at this season of the year, but sometimes the children's wards in the big institutions are a little overlooked, though these are really quite large hospitals in themselves. Over 150 small patients there are this Christmas in St. Thomas's Hospital, yet Sister Victoria has had to make special efforts to obtain a sufficient supply of dolls and toys, and the children's great Santa Claus, *Truth*, seems to have almost forgotten the existence of this particular children's ward. No large dolls have found their way into Victoria this year, and the *Truth* dolls of two years back have had to be re-polished and dressed to maintain the traditions of the ward, where, as in so many others, their advent is eagerly looked for, and a post of honour accorded to "the" doll of the year.

### Concert at the London Temperance Hospital.

It is the custom at the London Temperance Hospital to raise a special fund at Christmas time to defray the cost of the entertainments provided for the patients, the matron issuing an appeal for subscriptions towards this end. This year is no exception, and it is good to hear that Miss Lucas has received a satisfactory response so far. An excellent concert was given at the hospital on Saturday evening in aid of the fund, the whole programme being got up and carried through by Miss Constance May Hutton, who has given help of a similar kind on former occasions. The evening's entertainment went with great swing, and was thoroughly enjoyed by all who were present. Miss Hutton's own songs were worth a journey to hear, especially perhaps "A May Morning," while those of Mr. Arthur Thomas and Mr. Ben Griffiths met with much applause. Mrs. Royal-Dawson's recitations were excellent, and the duologue ("A Backward Child") between herself and her daughter, Miss Inka Royal-Dawson, particularly amusing. Miss Florence Hawkes played a "Scherzo" of Van Goens' on the violin, and later a solo on the mandolin. Piano solos were given by Miss Edith Greenop and Mr. A. Slater, the latter's rendering of Chopin's "Polonaise in A Flat" being especially fine. A duet, "In the Dusk of the Twilight," delightfully sung by Miss Hutton and Mr. Thomas, brought a very pleasant concert to a close. It was announced afterwards that the preparations for the entertainment had cost the hospital nothing; the piano was lent by Messrs. Erard; Miss Hutton herself defrayed the cost of the programmes; and the flowers and drapery for the platform were also kindly lent. The proceeds, which should be considerable, go, therefore, wholly to the patients' Christmas fund.

### West London Hospital.

Christmas is not kept to any great extent at this hospital. So many serious cases are admitted that it is impossible to have much excitement. Still the sisters manage to make the wards gay with evergreens and coloured ribbons, and Chinese lanterns, and the children's ward was especially bright with toys and glittering decorations that make the glory of Christmas trees. On Christmas Eve a voluntary choir, trained by Miss Richmond, visited the wards, and sang carols. One friend gave the Christmas puddings, another the tea to patients, nurses, and servants. On Monday night the nurses gave a little entertainment on their own account, which consisted chiefly of music and songs. Afterwards they were regaled with supper by the generosity of another friend, the wife of one of the visiting physicians. Christmas at West London Hospital was therefore a busy time to the nurses, and much enjoyed by all.

### Lectures on Medical Relief.

UNDER THE AUSPICES OF THE CHARITY ORGANISATION SOCIETY.

### THE SELF-SUPPORTING DISPENSARY AND DISTRICT NURSING ASSOCIATION.

THE last lecture of the series, given by Dr. Jamieson B. Hurry, M.A., late surgeon Reading Dispensary, &c., on December 9th, had for its subject the advocacy of "Provident Nursing Associations," established on a self-supporting, co-operative basis.

Dr. Hurry began by pointing out how common an occurrence is disabling sickness, statistics proving that during the working years of life, from fifteen to sixty-five, each person is on an average thrown out of work by illness for nine days per annum. The difficulty of dealing with sickness amongst the industrial classes was not to be met by hospitals, free dispensaries, free nursing institutions, or Poor Law relief, nor should the self-respecting working man be willing to resort to charitable institutions for the treatment of the ordinary ailments that occur in every family. The only satisfactory solution was to be found in adopting the principles of mutual assurance, already recognised so far as medical attendance was concerned by the establishment of provident dispensaries and clubs, but which should also be adopted in the case of nursing, since there was no more reason why skilled service should be given in the form of free nursing than in the form of free medical advice. Nursing was frequently as important as professional attendance, the doctor and the nurse being, in fact, twin forces, each of which apart from the other loses half its value.

Dr. Hurry deplored the spread of purely charitable relief, and the fact that the system of district nursing now springing up all over the country was, unhappily, based on charitable lines, of which the ultimate effect must be to injure the moral fibre of the people.

The lecturer then described a model organisation for supplying the wage-earning classes with both medical and nursing attendance on co-operative self-supporting lines. This should take the form of a combined dispensary and nursing institution, guaranteeing, in return for regular yearly payments, medical attendance, nursing, medicine, and the use of nursing appliances. There should be three conditions of membership: (1) Payment in advance; (2) an entrance fee, on a higher scale, in the case of people not on the roll of members; and (3) nomination by subscribers, at a cost to them equivalent to the entrance fee of Class 2, in the case of the necessitous poor. But the necessitous and improvident poor should, in Dr. Hurry's view, be dealt with by the poor law. The premium required from ordinary members of such an institution would vary according to local circumstances, but 2d. to 3d. a week would suffice to maintain it, when fairly afloat, on a self-supporting basis. The difficulty of starting the scheme could be met by a provisionary guarantee fund.

He thought that organisations established on these principles should be found in every centre of population, and would confer great and far-reaching benefits. By this scheme the working-classes would be able to ensure, on terms well within their means, both medical and nursing care in times of illness, and to pay a fair remuneration both to doctor and nurse. At the same time habits of thrift, self-reliance, and healthy independence would be fostered. By helping the industrial classes to help themselves, by bringing skilled medical and nursing attendance within their reach on terms within their means, and by raising the standard of comfort in the home, such institutions should powerfully promote social progress, national health, and the general welfare.

A very interesting discussion followed the reading of Dr. Hurry's paper, in which Mrs. Minet and others interested in district nursing took part, the general view clearly being that the scheme proposed might do well amongst the superior classes of working people, but was too Utopian for present establishment amongst the very poor, who as yet by no means realised the importance of trained nursing, and would seldom feel it necessary to pay for the services of the district nurse.

### To Pension Fund Nurses.

### MISS BURNS'S WEDDING GIFT.

WE publish below a further list of nurses who are contributing to the address to be presented to Miss Burns, daughter of the late Chairman of the Pension Fund, on her marriage. The contributions, which are limited to sixpence, are received by the Editor, The Lodge, Porchester Square, W. All names will be forwarded with the address, and this will be on show when completed:—H. R. Walker, Barkwith, H. A. Chaplin, P. A. Milne, Ayrton, M. Thomas, A. Warwick, C. E. Williams, S. Constantine, C. F. J. Marshall, E. Chapman, M. Barkley, F. Dell, M. Essex, S. H. Doughty, K. Prior, Clarke, Collingwood, Pexton, Doubell, Renwick, McEwan, Wood, B. Chapman, Empoon, E. Westlake, M. A. Mackie, A. R. Bellamy, F. Low, H. Elsby, E. M. Washtell, M. Welch, L. Gunn, M. Wood, A. E. Bodfish, C. Boulter, A Grateful Nurse, M. L. Smith, J. March, E. Osmond, Sister Gertrude, A. J. Little, Nurse Ewart, M. Brewster, A. M. Deville, A. Bates, F. I. Daniel, B. Kullor, A. L. Pratt, M. T. Nicholson, Howells, Bowlerwell, A. Cameron, Policy No. 1875, S. Watts, M. Stone, E. F. Graham, Chaplin, A. (Birkenhead), F. Cupit, C. Streeting, E. Boyd, R. Mason.

### Appointments.

BIRMINGHAM AND MIDLAND EAR AND THROAT HOSPITAL.—Miss Catherine Mary Archibald has been elected Matron of the above hospital. Miss Archibald has been connected with the Queen's Hospital, Birmingham, for nearly seven years. She was trained there, and successively passed through the various grades, finally becoming sister and taking charge of the various medical and surgical wards. There were sixty-eight applicants for this post.

BELPER ISOLATION HOSPITAL.—On the 16th inst. Miss H. A. Matthews was appointed Matron here. She was trained at St. Marylebone Infirmary, Notting Hill, London, and afterwards became charge nurse at the Borough Hospital, Eastbourne. She then served a year under the P. and O. Company, and for the last two and a half years has been sister at the Allt-yr-yn Hospital, Newport, Mon.

### Minor Appointments.

FEVER HOSPITAL, THORPE, EASINGTON, DURHAM.—Miss Lucy Osborne was appointed Matron here on the 15th inst. She was trained at the Chichester and Preston Royal Infirmary, and she has been nurse at Mill Lane Hospital (Wallasey Urban District Council), nurse at the Infectious Diseases Hospital, Farnworth, near Bolton, and charge nurse of the Cottage Hospital, Mold.

ST. SAVIOUR'S UNION INFIRMARY, S.E.—Miss Agnes Gardiner, who was trained at the Royal Infirmary, Liverpool, has been appointed Night Superintendent of the female wards here. She has been "Sister" at Carnarvon Infirmary and at Isleworth Infirmary.

### "THE HOSPITAL" CONVALESCENT FUND.

OUR thanks are due to Miss Hotson for her kind contribution of 2s. 6d. to the above fund.

### Everybody's Opinion.

[Correspondence on all subjects is invited, but we cannot in any way be responsible for the opinions expressed by our correspondents. No communication can be entertained if the name and address of the correspondent is not given, as a guarantee of good faith but not necessarily for publication, or unless one side of the paper only is written on.]

### OUR CLOTHING DISTRIBUTION.

MISS MONK, King's College Hospital, writes: Please accept our most grateful thanks for the parcel so kindly sent for our poor patients this Christmastide, and which will be most useful.

THE SECRETARY, Charing Cross Hospital, London, W.C., writes: I am directed by the Board of Governors to convey to you their grateful thanks for your kind present of clothes for the patients.

THE MATRON, West London Hospital, Hammersmith, W., writes: Thank you so much for the kind present of clothes you gave us from the "Hospital Clothing Distribution." Everything will be most useful and much appreciated by the patients.

THE MATRON, Westminster Hospital, Broad Sanctuary, S.W., writes: I beg to thank you very warmly for your kind and most acceptable gift of clothing for our patients. I can assure you these will prove most useful, for our demand for such things is sometimes far greater than our supply.

THE MATRON, the North-Eastern Hospital for Children, Hackney Road, Shoreditch, writes: The pretty frocks, crossover, and other articles of clothing will be most useful in the wards, and I heartily thank you and the kind contributors for such a helpful addition to the children's wardrobes.

THE MATRON of the Alexandra Hospital, 34, Guildford Street, writes: Please convey our very best and warmest thanks to the kind nurses who have spent so much time in making us such useful and good clothing. We are all very grateful indeed. It much surprised our children, who are old enough to consider, that the nurses should have found time to complete so much work. They are sure they do it "off duty," which "makes it ever so much kinder," they added. We all value such a handsome gift very much, and again thank our kind friends.

### HOSPITAL HOUSEKEEPING.

"A CORRESPONDENT" writes: I was much obliged to you for inserting the reply to my question as to "the kinds of fish as cheap as cod." The result of my writing to the fish merchant is that he tells me plaice is generally three times the price of cod. Letters on housekeeping are most valuable, and many officials who have to undertake that branch of work have, as your correspondent of this week states, no time to go about and try where anything may be cheapest at the moment, therefore any information is acceptable. I have found that children of poor people prefer rice pudding to any other form of milk pudding; and when, years ago, I trained in a London hospital, on asking why the milk pudding was never varied, I was told that more patients ate rice than puddings of any other grain.

### A HOME FOR NURSES NO LONGER ABLE TO WORK. —AN APPEAL TO PUBLIC OPINION.

MISS B. C. DAVIS, Bromley Cottage Hospital, Kent, writes: It must have struck all who are interested in nursing that among the multitude of institutions set apart for those whose means are limited there is no such home for nurses. There are several for governesses, clergy widows, &c., whose incomes are small, many for the poor and working class, but not one for those who devote their lives—at any rate the best years of their lives—to the relief of the sick and suffering, and who at the end of their years of activity find themselves without the means of affording such comforts as their early training, education, and mode of life have made them accustomed to expect. Nurses through no fault of their own are frequently left at a comparatively early age with savings or a pension barely sufficient to support existence. Their incomes, unless they get the plums of the profession, are small, £25 to £30 being their average salary. Those who are private nurses, taking two guineas a week, if they work all the weeks of the year only earn £100 or a little over, and out of this money they must provide for times when they are not at a case, for telegrams, cabs, uniform, and, as I say, a certain amount of board and lodging. They frequently have no private means. Still more often do they help their own families with their earnings, and this renders it impossible for a great number to avail themselves of the benefits of the Royal National Pension Fund. To secure an annuity of £30 a year at the age of 50 a nurse beginning at 21 must pay from £10 to £12 a year. At London hospitals nurses do not begin their training until 23 or 25 years of age. During this training they earn £16 or £18 per year—a limited number may even have to pay premiums. Thus during their training they are not in a position to pay towards the Pension Fund. Then by the time they are 25, which is the time most nurses begin to pay into the fund, it is necessary for them to pay over £14 a year, and if they wait until they are 30 years of age they have to pay £19 a year. Clearly this is impossible for most nurses, whose outside wage is £30. And, again, if even a nurse affords the premium, the income resulting only affords a bare existence. The ranks of the nursing profession are so largely recruited from the professional classes that to be forced to live on £30 a year is for many a real hardship. Let those who shield their own daughters from every breath of discomfort think what it would be to those girls if through loss of money they in years to come must board, lodge, dress, &c., on £25 or £30 a year. Therefore I cannot help thinking that for those nurses a home affording permanent lodging rent free would be an inestimable boon. Details would have to be arranged by a committee according to funds, but the rough outline of the scheme is a house or houses, rent and taxes paid, if possible lights and coals provided, the nature and extent of the accommodation to be considered by the committee of management. A resident superintendent, who would also be secretary, would be necessary. She would be responsible for seeing to any small repairs, for interpreting and enforcing rules (which ought to be as few as possible), for receiving all applications and sifting the same in order to lay before the committee all necessary information regarding the candidates. These candidates would be selected by the committee, and a careful discrimination between individual cases, apart from the general rules concerning age, income, and length of service, would be a pressing necessity. Such a home should be built a short distance from London—for nurses within the metropolitan area—to accommodate 25 or 30 nurses. The numbers could be increased if the funds subscribed admitted of it, and additions could be made to the accommodation at any time subsequent to the establishment of the institution. The buying of land, building, and endowment of the same would take about £50,000. If an institution of this kind were established for London probably other large towns would follow suit. So much has been done of late for hospitals that sympathy will surely be forthcoming for those who work for and in hospitals. It would be a reproach to London if all those who spend health and strength in the service of the sick in its midst were not fully considered and helped. I cannot but think that if the scheme were widely known it would meet with sympathy and response, and I would ask all who read this article to interest themselves and their friends. Any suggestions would be gratefully received by the writer.

### Presentations.

MISS DUNCAN, superintendent of the Blackheath and Richmond Nursing Institutions, was presented at Christmas with a beautiful "Queen Anne" teapot by a large number of her staff.

MRS. RHODES, matron of the Nurses' Home, Catherine Street, Liverpool, was presented with a handsome davenport and a water-colour drawing as a Christmas gift by the nurses connected with the home.

### Wants and Workers.

"E. N. S.," care of H. W. Steel, Talgarth Hall, Machynlleth, North Wales, would be glad to obtain an invalid tricycle, to be worked with the hands and not with the feet. It is for a woman who cannot use her legs.

### For Reading to the Sick.

THE WANING YEAR.

**Verses.**

THE year departs! a blessing on its head!
We mourn not for it, for it is not dead.
Dead! What is that? A word to joy unknown,
Which love abhors and faith will never own.
The passing breezes, gone as soon as felt,
The flakes of snow that in the soft air melt,
The smile that sinks into a maiden's eye,
They come, they go, they change, they do not die.
So the Old Year—that fond and formal name,
Is with us yet—another and the same.
And are the thoughts that ever more are fleeing,
The moments that make up our being's being,
The silent workings of unconscious love,
Or the dull hate which clings and will not move,—
Are these less vital than the wave or wind,
Or snow that melts and leave no trace behind?
—*H. Coleridge.*

Now it is gone. Our brief hours travel past
Each with its thought or deed, its why or how,
But know each parting hour gives up a ghost
To dwell within thee—an eternal now.
—*S. T. Coleridge.*

Have I laid by from summer hours
Ripe fruits as well as leaves and flowers?
Hath my past year a growth to harden,
As well as fewer sins to pardon?
Is God in all things more and more
A king within me than before? —*Faber.*

No action, whether foul or fair,
Is ever done, but it leaves somewhere
A record written by fingers ghostly
As a blessing or a curse, and mostly
In the greater weakness or greater strength
Of the acts which follow it—till at length
The wrongs of ages are redressed
And the justice of God made manifest.
—*Longfellow.*

In doing is this knowledge won,
To see what yet remains undone.
Within this our pride repress,
And give us grace, a growing store,
That day by day we may do more
And may esteem it less. —*Trench.*

Life to come will be improvement on the
life that's now! Destroy
Body's thwartings—there's no longer screen betwixt
Soul and Soul's joy. —*Browning.*

Our many thoughts and deeds, our life and love,
Our happiness and all that we have been,
Immortally must live and burn and move. . .
Fate—Time—Occasion—Chance—and Change: to these
All things are subject but Eternal Life.
—*Shelley.*

**Reading.**

We must learn to detach ourselves from all that is capable of being lost, to bind ourselves absolutely only to what is Absolute and Eternal. . . There is no repose for the mind except in the Absolute; for feeling, except in the Infinite; for the Soul, except in the Divine. The Ideal after all is truer than the Real; for the Ideal is the eternal element in perishable things.—*Amiel.*

There is no separation—no Past; Eternity—the Now—is continuous. . . The continuity of now is for ever.—*Richard Jefferies.*

**Prayer for the Week.**

We have followed too much the devices and desires of our own hearts. We have left undone those things which we ought to have done, and we have done those things which we ought not to have done, and there is no health in us. But Thou, O Lord, have mercy upon us! Forgive us all that is past, and grant that we me may ever hereafter serve and please Thee in newness of life.

### Death in Our Ranks.

WE regret to chronicle the death of Nurse Skipp, after a short illness contracted in discharge of her duty as charge nurse at the West Ham Infirmary. Nurse Skipp went as probationer to Lambeth in 1895, and received her certificate of training and promotion at the end of her three years' course. In October of this year she was appointed charge nurse at the West Ham Infirmary, where, in nursing a case of typhoid, she caught the infection and died. Her kind and gentle disposition had won much respect from her patients and love from her fellow nurses, three of whom (as representatives of the Lambeth staff) attended her funeral, bearing with them most beautiful floral tokens to be laid upon the coffin as a tribute of affectionate regard to the memory of the deceased.

### Notes and Queries.

**The contents of the Editor's Letter-box have now reached such unwieldy proportions that it has become necessary to establish a hard and fast rule regarding Answers to Correspondents. In future, all questions requiring replies will continue to be answered in this column without any fee. If an answer is required by letter, a fee of half-a-crown must be enclosed with the note containing the enquiry. We are always pleased to help our numerous correspondents to the fullest extent, and we can trust them to sympathise in the overwhelming amount of writing which makes the new rules a necessity.**

**Every communication must be accompanied by the writer's name and address, otherwise it will receive no attention.**

*Spinal Disease.*

(141) Can you kindly tell me of an inexpensive book on spinal disease (tubercular)?—*Rose.*

The subject of tuberculous disease of the spine is a part of general surgery, and is decribed in the surgical manuals.

*Superannuation Acts.*

(142) Will the Editor kindly tell me what is meant by the term, "Subject to the Poor Law Officers' Superannuation Acts," or where such information can be fully obtained?—*Constant Reader.*

It means subject to a law enabling the Poor Law authorities to keep back a portion of the salary or wages of those employed under them, so as to provide a fund from which those employed are granted pensions after having reached 60 years of age. A copy of the Act may be obtained from any law stationer for a trifling sum.

*Catheter.*

(143) Where can a partly-trained nurse be taught catheter work in London?—*Nurse Florence.*

Ask the district nurse to teach you, or the medical man for whom you are working.

*The Law of Notice.*

(144) Will you kindly inform a "District Nurse" who was engaged a year ago by a committee, paid weekly, if they can now make a rule compelling the nurse to give three months' notice if at any time she wishes to leave? Length of notice was never mentioned when she was engaged.

The ordinary rules would hold good in absence of any specified agreement, namely, weekly wages, weekly notice. It is quite possible, however, for the committee to make new regulations, and to ask the nurse to accept them; if she declines, which she has a right to do, they can give her a week's notice and engage someone else.

*Distressed Nurses.*

(145) Kindly tell me if there is any charity for helping distressed nurses in ill health who have not been able to pay pension fund premiums. I have been a district nurse, holding my last post just over two years. I have been in ill-health, and have rested now for six months without regaining my health. I am in straitened pecuniary circumstances. I find many nurses are widows with children dependent on them, and therefore are not able to pay pension fund premiums. So that when broken down in health the anxiety about the future greatly retards their convalescence.—*E. J. B.*

A nurse is eligible for help from all charitable agencies for women in her circumstances (see "Burdett's Hospitals and Charities," Scientific Press, London, W.C., 5s.). She is also eligible for the "Trained Nurses' Annuity Fund," of which Mr. R. Gofton-Salmond, 72, Cheapside, E.C., is the secretary. Nurses who are members of the Royal National Pension Fund enjoy the benefits of the Junius Morgan Benevolent Fund, and trained nurses who only require rest to enable them to resume their duties are helped by the "Hospital Convalescent Fund," particulars of which will be sent on application to the Hon. Secretary at this office.

*Falling Hair.*

(146) In an issue of THE HOSPITAL there appeared an article upon human hair, giving the reason of falling off and baldness, and recommending a remedy to be obtained at a chemist's in New Bond Street, London. The name of the writer, a doctor, as well as the name and address of the chemist, I have entirely forgotten, and, unfortunately, I cannot lay my hands on the number of THE HOSPITAL containing it. I would take it as a great favour if you could assist me in the matter.—*District Nurse.*

The article to which you refer is entitled "Dandruff and Baldness," and is by Dr. Leslie Phillips, of Birmingham. It appeared in THE HOSPITAL of July 31st, 1897. The ointment spoken of by him—Behrendorf's Pomade Pongrée—is supplied by Messrs. May, Roberts, and Co., 9 and 11, Clerkenwell Road, E.C. The treatment of dandruff, however, does not consist merely of the application of an ointment.

### Travel Notes.

By Our Travelling Correspondent.

Rules in regard to Correspondence for this Section.—All questioners must use a pseudonym for publication, but the communication must also bear the writer's own name and address as well, which will be regarded as confidential. All such communications to be addressed "Travel Editor, 'Nursing Mirror,' 28, Southampton Street, Strand." No charge will be made for inserting and answering questions in the inquiry column, and all will be answered in rotation as space permits. If an answer by letter is required, a stamped and addressed envelope must be enclosed, together with 2s. 6d., which fee will be devoted to the objects of the "Hospital Convalescent Fund." Any inquiries reaching the office after Monday cannot be answered in "The Mirror" of the current week.

### IV.—THE RIVIERA : NICE.

Hotels in Nice are very expensive, and it is well to grasp the unpleasant fact at once ; living altogether is a costly affair whether in hotels, pensions, or apartments. These latter, however, if taken unfurnished and for the entire year are sometimes to be had on very reasonable terms, so much so indeed, that it is worth while to do it, if one member of a family is compelled to winter abroad every year ; the loss sustained in the summer by having the house unoccupied is counter-balanced by the enormous saving effected during the expensive season. I have friends who do this and who tell me they distinctly gain by such an arrangement.

### Hotels and Pensions.

Their name is legion, and some of them are reasonable, but living is expensive, and therefore it is impossible that hotel proprietors any more than anyone else can give really good food and accommodation on low terms. If your means admit of it, it is always delightful to have a window looking on the Promenade des Anglais, but that means at the lowest 18 to 20 francs a day ; the Hotels des Anglais, Rome, Westminster, Méditerranée, &c., all on this celebrated esplanade, vary but little in charges, and for very small rooms somewhat high up I fear you must reckon your expenses at 18 francs a day. They announce pension terms from 14 to 20 francs, but 14 would mean a minute apartment at the top of the house, and looking to the back, all which, for an invalid, would be inadmissible. In the matter of hotels, it would be best if you are undecided, to let me know in good time your requirements, the state of the invalid, and the amount that can be spent each day or week, otherwise information on the subject is apt to be too vague to be really helpful. The Hotel Pension Suisse is excellent, and very much more reasonable, but it is very popular and almost always full, therefore application for rooms must be made some time in advance, or disappointment may occur. The aspect is the same as that of the houses on the Promenade des Anglais, but it is at the extreme end of Nice, under the Castle, and near to the Port. I should avoid hotels in the Carabacel quarter, because though somewhat cheaper, it is far from the sea and not convenient for shops, &c. At the same time, it is very sheltered and warm. If "money is no object," I should choose the Mount Boson Hotel, which is placed high in an ideal situation on the side of Nice, towards the exquisite bay of Ville Granche (Villa Granca). Another favourite hotel of mine is the Villa Arson. It is a charming place in which to stay, an old Italian villa with beautiful paved terraces leading up and down, magnificent palms half-veiling the curious seventeenth century statuary, and surrounded by rich olive woods, carpeted in the spring by purple iris and other lovely flowers. I shall give a sketch of this enchanted spot next week. It stands above the village and monastery of St. Barthélemy, and though some distance from Nice it is by no means isolated. A train runs every 20 minutes from St. Barthélemy, and the hotel omnibus makes the *trajet* frequently.

### General Characteristics of the Town.

Hare calls Nice an "ugly, modern town, with Parisian shops and a glaring esplanade along the sea." I cannot agree with him. It is true as to its being modern, but surely not ugly. The main street of shops, called the Avenue de la Gare, is very striking and pretty, with trees planted each side, so that they meet overhead and make a delightful shade in the burning spring sun ; every small side street discloses views of the distant hills clad in every hue of the opal, and as for the Promenade des Anglais, upon which he is so severe, modern though it be, it is one of the joys of Europe.

A Corner of Old Nice.

### The Promenade des Anglais.

What a pleasure for an invalid whose exertions, perhaps, are limited to five minutes' walk to be able to step from his hotel on to this glorious sea-front, to sit basking in the life-giving sun from morn till eve, and gaze upon the sapphire and emerald sea that never remains the same for ten minutes together. To the left rises the promontory crowned with the ruins of the old citadel, and to the right are seen the fairy Esterelles, always more or less veiled in mist. The Esterelles, considered from the point of view of a sketch, have proved too hard a nut for some artists of no mean repute to crack, so I refrained from attempting it, though the temptation was great. Alas! one needs a fairy brush and ethereal colours to portray that delicate loveliness. As the Promenade des Anglais widens out at the Jardin Public, one comes upon the band stand—plentiful shade and numerous seats make this a delightful lounge all day; it is easy to carry a deck chair with you, and still paying a sou for one for the occupier's feet, so as not to affront the *droits de place*, your invalid can remain all day working, sleeping, reading, and listening to the band, and thus drink in life with the warm, lovely air of that incomparable promenade. If you quarrel with its undeniable modernity and Philistine comfort, rest happy, there is still plenty that is picturesque and inconvenient in the old town.

### The Old Quarter of Nice.

This lies in the part extending from the Place Messena to the charming old Port, where all kinds of craft are anchored between their voyages to and fro to the different Levantine ports, to Marseilles and Corsica. The most artistic corners are to be found round the old Cathedral of S. Reparata, of which I give you a sketch. There is a market for fruit and vegetables held under the shadow of the south side, which is one of the busiest scenes conceivable up to midday; the awnings of every shape and colour, agreeably variegated with adorable patches, and the stalwart dames and brigand-like men who tout their wares below have a most taking appearance. The women usually have gay handkerchiefs crossed over their heads, emerald green and orange being the favourite colours; and the men wear a species of scarlet jelly bag dangling over one ear that seems the peculiar property of that seaboard. These gentry speak the Niçois patois entirely incomprehensible to outsiders; it resembles Italian more than French—just as the natives themselves cling fondly to the memory of their relation to Italy. It has only become French territory within the last 40 years, and the old Niçois still resent the change. The artist will find plenty of field for sketching about here, and I am happy to record the fact that it is freer from noisome odours than most picturesque old towns. The intense power of the Southern sun caused the old builders to make their streets very narrow, and they are further shaded by acres of awnings spread over the open shop fronts, making a most artistic confusion of brilliant light and sombre shadow. The old town climbs higher and higher up the sides of the steep hill crowned with the castle ruins like an advancing tide, and it is on this side that the architectural artist will find many taking little scenes.

### The Price of Carriages.

This is not exceptionally high, though dearer than in places less frequented by the English. A barouche and pair can be had for the entire day for 30 francs, and I need hardly remind you there is no better cure for many invalids in the earlier stages of consumption than life in the open air without fatigue. I have known some wonderful cases of complete recovery from this treatment alone, and no place can be better adapted for carrying it out than Nice. Its environs are unsurpassed for variety and beauty. In itself, as a town, it is not nearly so pretty as Mentone or San Remo, but as a centre it is more conveniently placed. By degrees I shall tell you of the charming places hidden in the lateral valleys and on the wonderful *Chemin de fer au Sud*, which is like a railway through fairy land. If your invalid is not seriously ill and money is an object, you can explore these lovely spots very cheaply by the help of this remarkable railway and by the innumerable diligences that ply in and out of Nice in all directions. I have never been blessed with much of this world's gear, all the same I know nearly every inch of the enchanting Riviera far better than most of my wealthier sisters. Still, I am well aware that this argues a certain amount of robust health, but then I am not only writing for invalids, but also for their friends and attendants. In consequence of the proximity of Monte Carlo, the train service on the main line is excellent; everything is done to make things easy for those who risk the smiles of fortune, and so one can get to all the principal places along the sea coast very easily, cheaply, and with no fatigue, though it is naturally far more delightful to drive along the Corniche. There are now two roads bearing the same name, the higher and original one and another low down close to the railway. It is well to go to any given spot by the higher and return by the lower, which is much more sheltered.

### Churches in Nice.

There is an excellent English church, far beyond the usual provision for our spiritual needs on the Continent; it is fairly large and always very full. The service is well and reverently done in a simple way and the chaplain appears to be a popular man. There is also an American church where the music is extremely good in a congregational way. In the Avenue de la Gare stands the modern Roman Catholic church of Notre Dame, very fashionable and crowded, and for those who like to worship among the time-honoured stones and pictures of the past there is the old Cathedral of St. Reparata.

### Tram Lines.

One set of trams start from the railway station at Nice, and going straight through the town by the Place Messena draw up at the old Port; others go in opposite directions, always touching on the station line, which is in every way a convenience. There is hardly any part of Nice from which you cannot reach the station without walking. An electric tram starts every half-hour from the centre of the town, taking passengers to Cimiez; it is agreeable to go up in this way (for it is a steady pull) and to walk the return journey. Cimiez, which may now be considered a suburb of Nice, has become very popular since our Queen has stayed there. Two monster hotels somewhat block the views, but the air is most invigorating, and for some cases makes it a most desirable residence. The old Franciscan convent with its remarkable giant ilexes and singular crucifix, with its six-winged seraphim, is full of interest for artists. There is a curious subterranean passage in this neighbourhood passing under the Paillon; there is a weird entrance to it near the Franciscan convent, but the frightful noise of its mysterious water in no way tempted me to explore it, but I believe adventurous spirits may do so. Hare gives a singular legend connected with this dismal hollow, in which the Devil and a golden goat and kid play a prominent part.

## Figures and Tables

**Figure f1:**